# Endoscopic submucosal dissection for high-risk lesions in the right colon: Limited benefits and significant challenges

**DOI:** 10.1055/a-2543-1484

**Published:** 2025-03-17

**Authors:** Francesco Vito Mandarino, Julia L Gauci, Sunil Gupta, Nicholas Burgess, Michael J Bourke

**Affiliations:** 1 8539 Department of Gastroenterology and Hepatology, Westmead Hospital, Sydney, Australia; 2 522555 Medicine, The University of Sydney Faculty of Medicine and Health, Sydney, Australia



We read with great interest the paper by Alfarone L et al., which evaluated outcomes of endoscopic submucosal dissection (ESD) for right colonic large non-pedunculated colorectal polyps (LNPCPs) at high-risk of submucosal invasive cancer (SMIC) 1 . The authors reported an en bloc resection rate of 84.5%, an R0 resection rate of 78.4%, and a curative resection rate of 72.4%, with adverse events (AEs) occurring in 19% of cases. They concluded that ESD is effective and safe in this cohort.

Although a selective ESD strategy for high-risk SMIC lesions in the right colon appears more promising than a universal ESD approach, a deeper interpretation of the outcomes is needed to assess the true risk-benefit ratio of this strategy.

The study reports T1 adenocarcinoma in 29 lesions (25%); however, curative resection was achieved in 12 cases (10.3% of the cohort). Even in expert hands, only 65% of SMIC cases were resected R0. In a recent multicenter study, the rate of right colon LNPCPs with low-risk cancer was 0.78% (23/2940) 2 and 17% (4/23) of them were managed en bloc with endoscopic mucosal resection (EMR). Applying this rate to Alfarone’s cohort, only 10 of 116 cases (8.6%) would have required ESD and not piecemeal resection to achieve cure (number needed to treat to avoid one surgery: 12). Further refinement of the current algorithm may be required. For example, nodule size of 10 mm in a granular lesion is a suitable threshold in the rectum but too small for the right colon, where risks of covert SMIC are much less 2 .

The authors report an intraprocedure perforation rate of 9.9%, which is notably higher than for EMR. Although no patients required surgery, this may not reflect real-world outcomes. A meta-analysis of 14,584 colorectal lesions treated with ESD found that surgery for AEs was required in 3.1% at Western tertiary centers 3 .

ESD is significantly more costly than EMR. In a previous study conducted over an 18-month period to account for late surveillance as mandated in the pre-margin thermal ablation (pre-margin thermal ablation [MTA]) era, a universal ESD strategy incurred costs of $6.91 million per 1,000 cases compared with $4.22 million for wide-field EMR 4 . Cost savings from EMR are likely to be substantially greater in the contemporary era where MTA has dramatically reduced recurrence and negated the requirement for a second (late) surveillance colonoscopy because delayed recurrence is now vanishingly rare. Alfarone L et al. report 3.1% early recurrence post-ESD, which exceeds that seen with EMR in the MTA era (< 2%) 5 .

The authors should be congratulated for investigating outcomes of a selective strategy by identifying higher-risk lesions that are more likely to benefit from en bloc resection. However, the low rate of curative resection and relatively high frequency of intraprocedure perforation suggest that caution is warranted in recommending an ESD strategy for high-risk lesions in the right colon. This paper demonstrates that the limited oncological benefits may not outweigh the significant resource demands, technical challenges, and higher risk of AEs incurred by ESD.

**Publication note**

Letters to the editor do not necessarily represent the opinion of the editor or publisher. The editor and publisher reserve the right to not publish letters to the editor, or to publish them abbreviated or in extracts.
